# Supplementary figures and images for: Transcriptome-wide identification of 5-methylcytosine by deaminase and reader protein-assisted sequencing
Source: eLife. 2025 Apr 8;13:RP98166. doi: 10.7554/eLife.98166 (PMC11978299; doi:10.7554/eLife.98166)

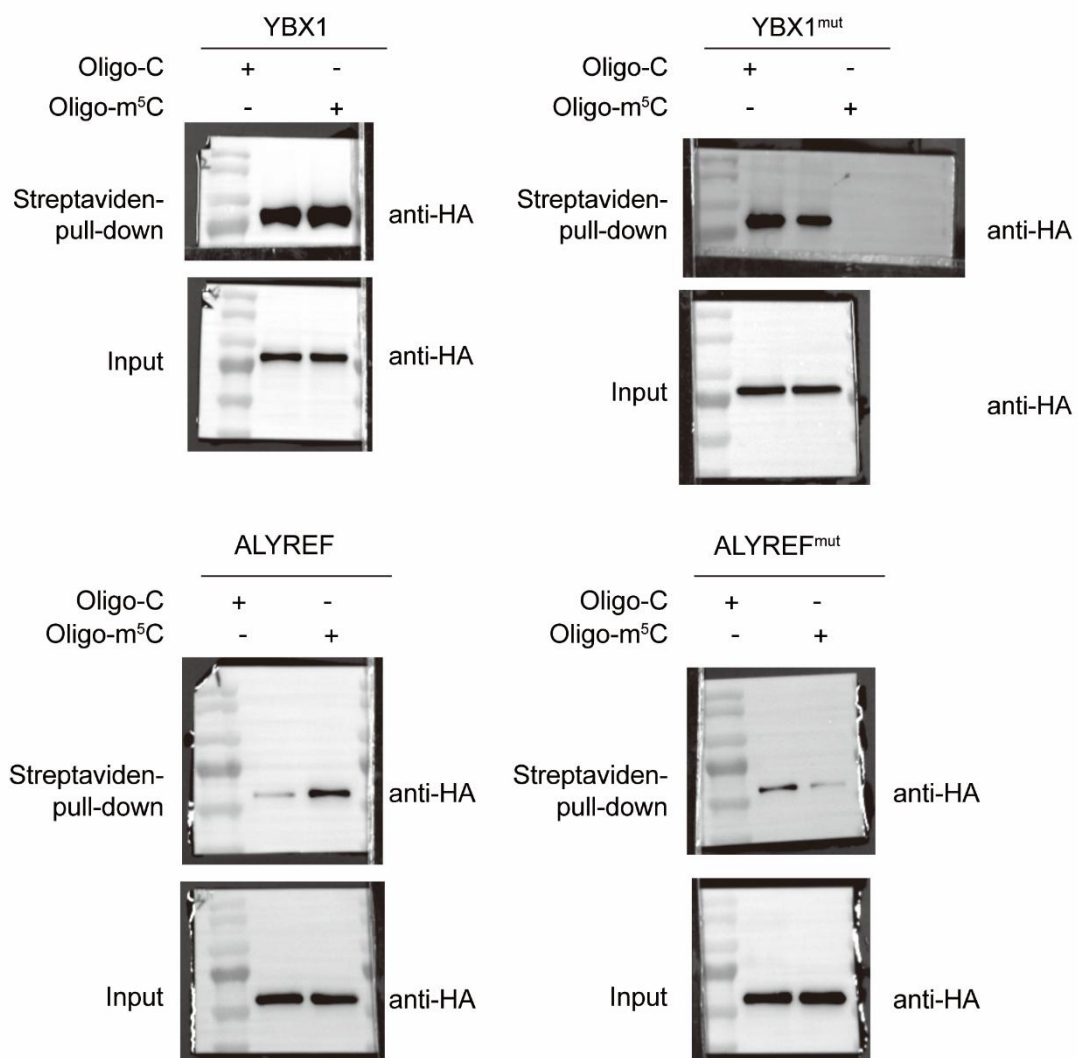

Supplement: Figure 1—figure supplement 1—source data 1. [file elife-98166-fig1-figsupp1-data1.zip › Figure 1-figure supplement 1-source data1/Figure 1-figure supplement 1-source data1.pdf]

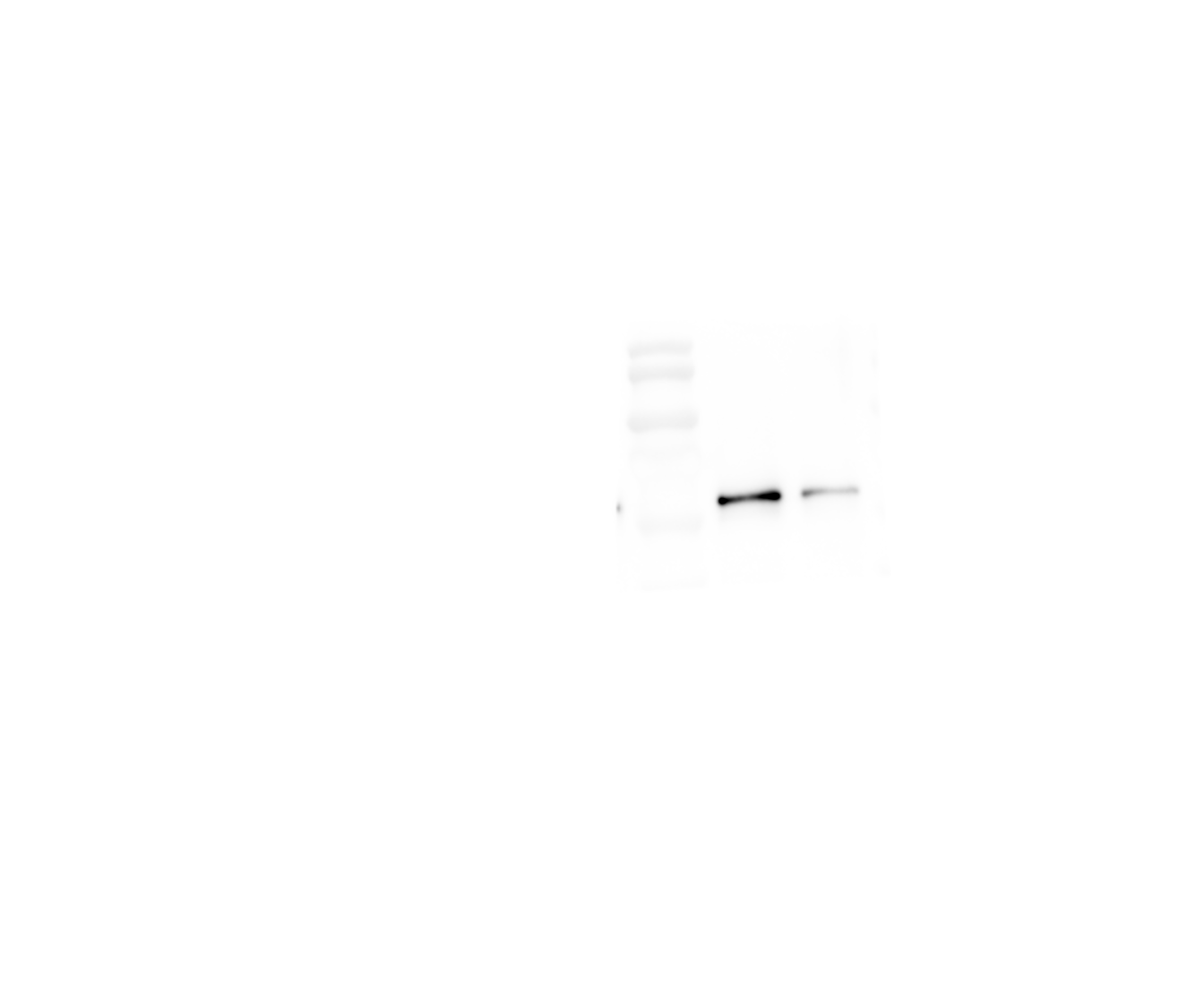

Supplement: Figure 1—figure supplement 1—source data 2. [file elife-98166-fig1-figsupp1-data2.zip › Supplementary Figure1H-pull-down (HA)-source data.Tif]

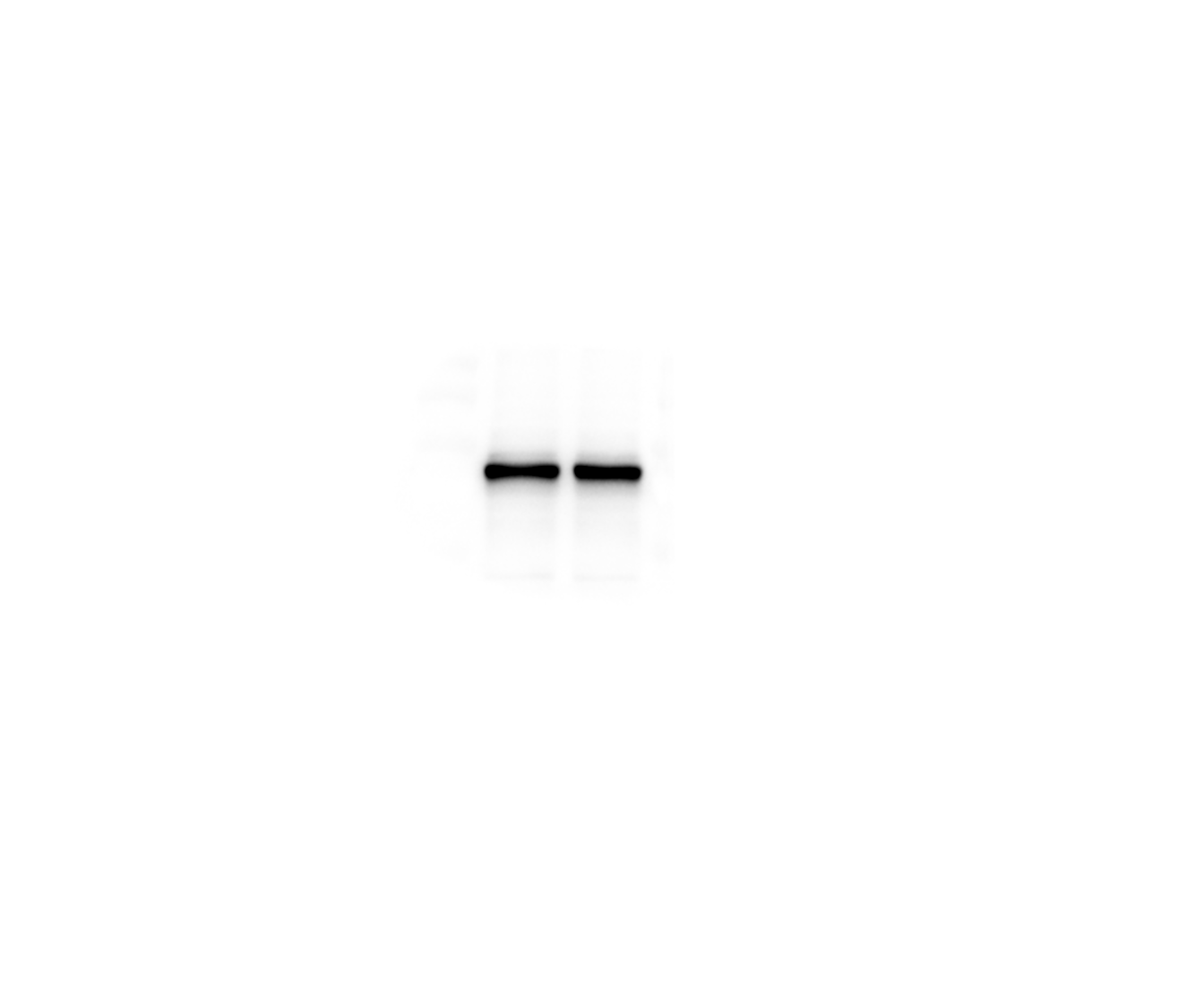

Supplement: Figure 1—figure supplement 1—source data 2. [file elife-98166-fig1-figsupp1-data2.zip › Supplementary Figure1E-input∩╝êHA∩╝ë-source data.Tif]

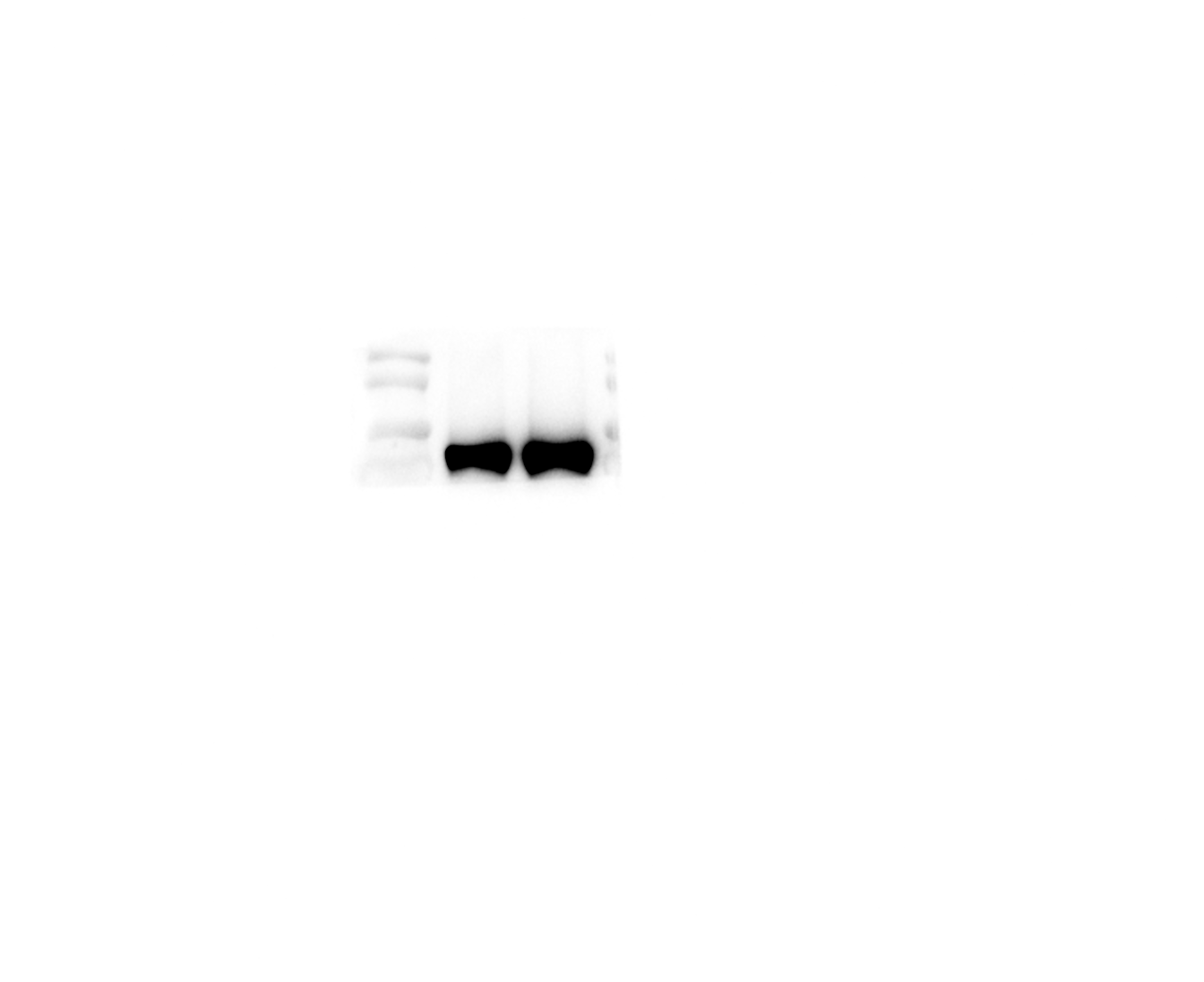

Supplement: Figure 1—figure supplement 1—source data 2. [file elife-98166-fig1-figsupp1-data2.zip › Supplementary Figure1E-pull-down (HA)-source data.Tif]

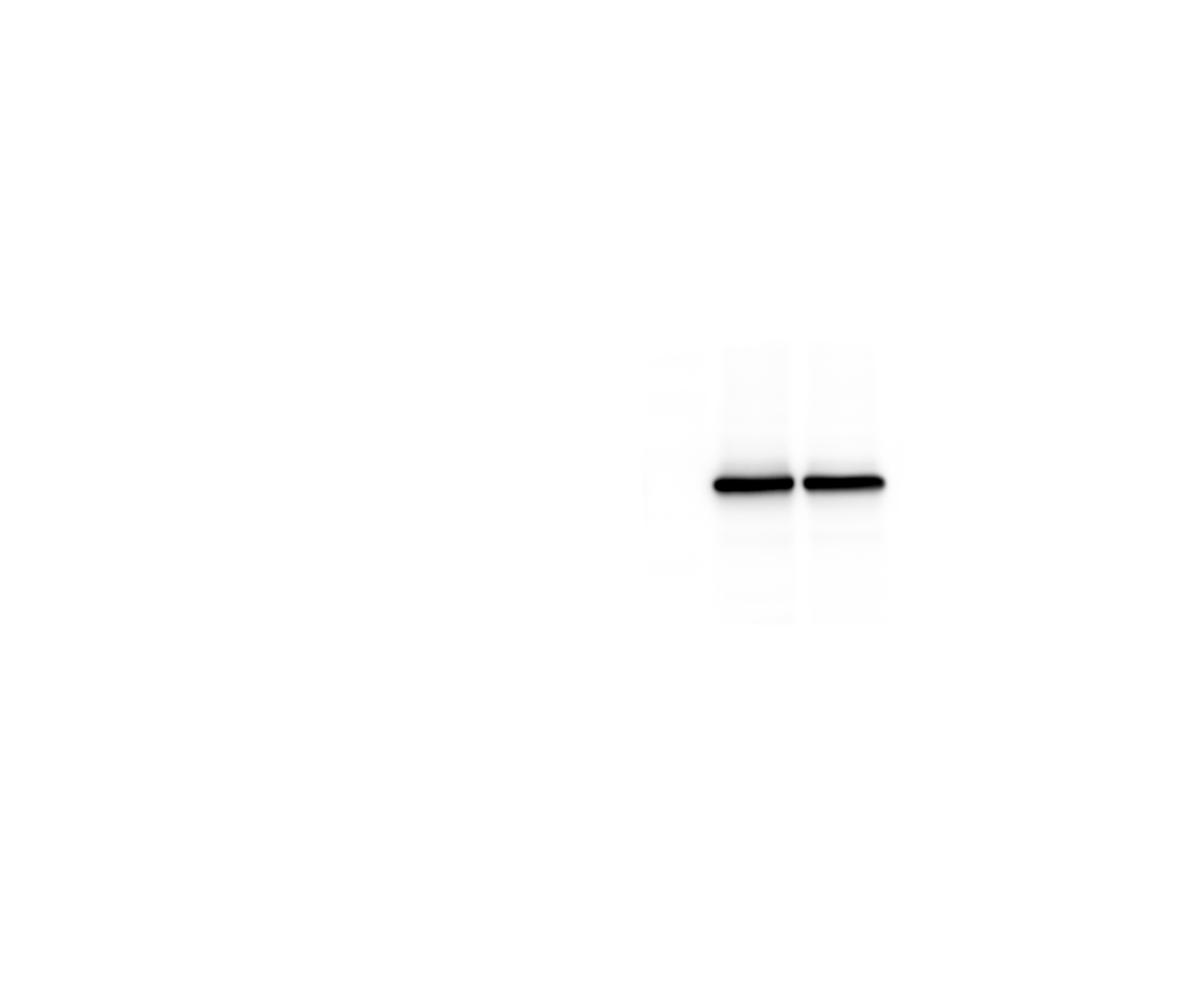

Supplement: Figure 1—figure supplement 1—source data 2. [file elife-98166-fig1-figsupp1-data2.zip › Supplementary Figure1F-input∩╝êHA∩╝ë-source data.Tif]

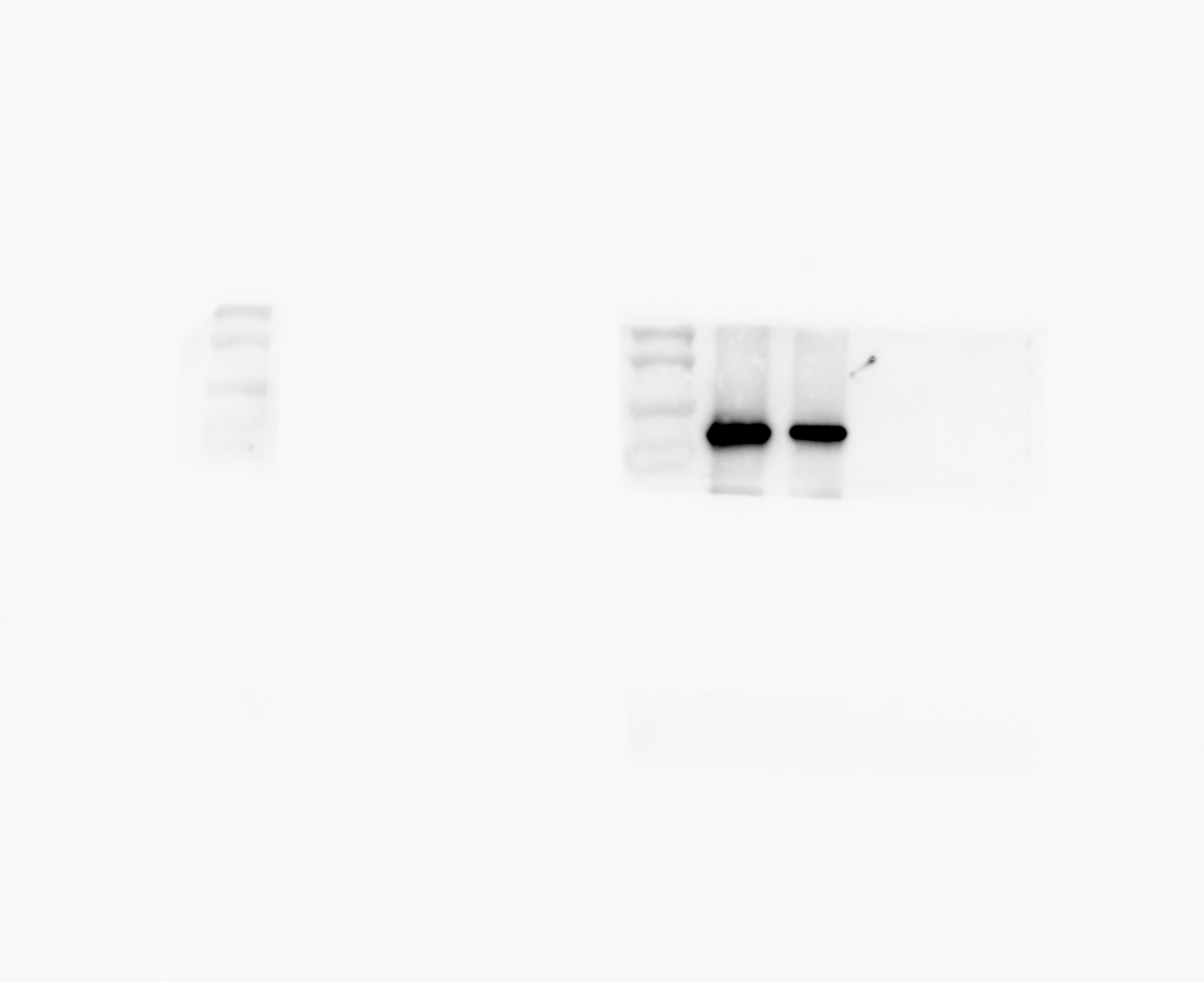

Supplement: Figure 1—figure supplement 1—source data 2. [file elife-98166-fig1-figsupp1-data2.zip › Supplementary Figure1F-pull-down (HA)-source data.Tif]

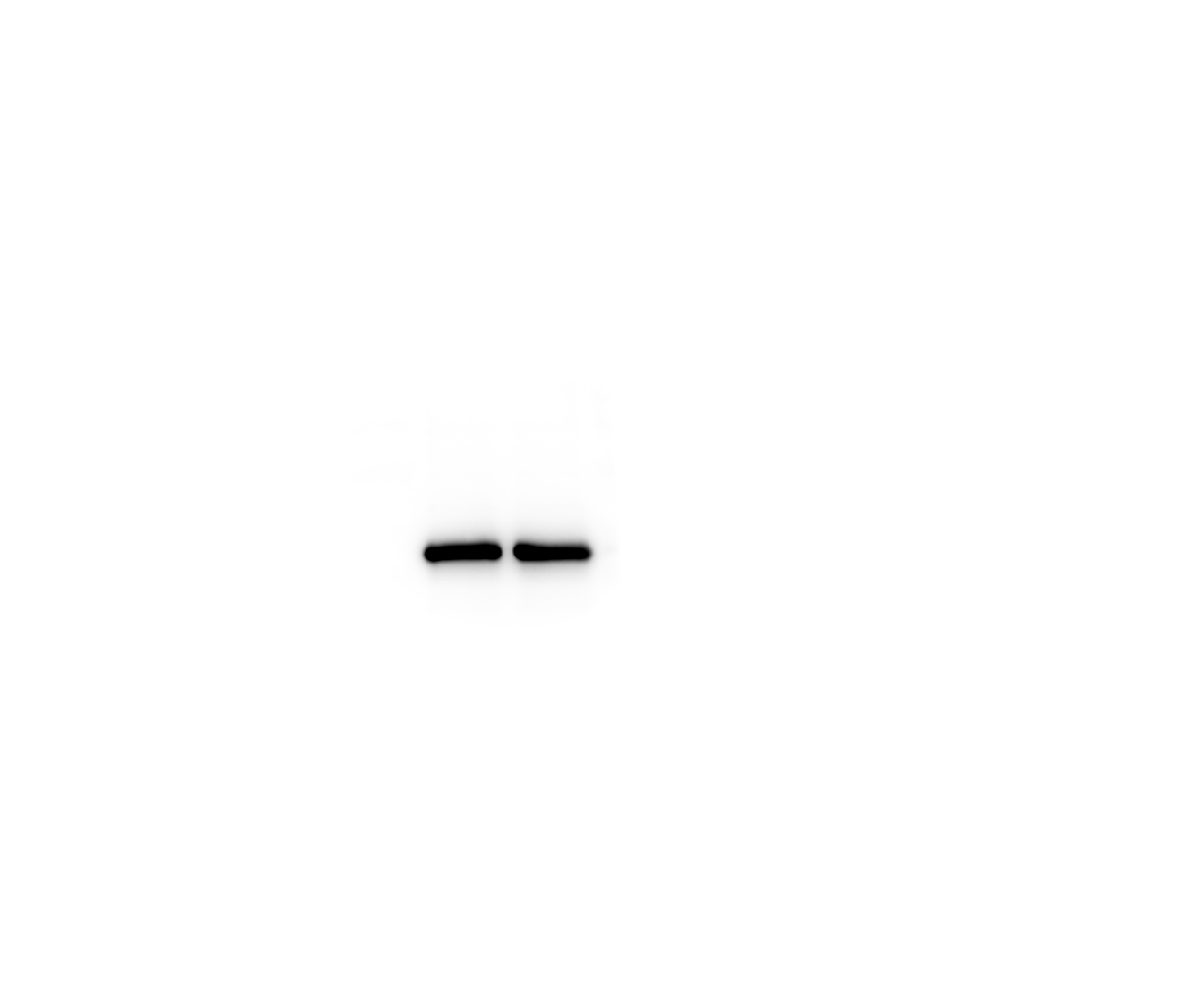

Supplement: Figure 1—figure supplement 1—source data 2. [file elife-98166-fig1-figsupp1-data2.zip › Supplementary Figure1G-input∩╝êHA∩╝ë-source data.Tif]

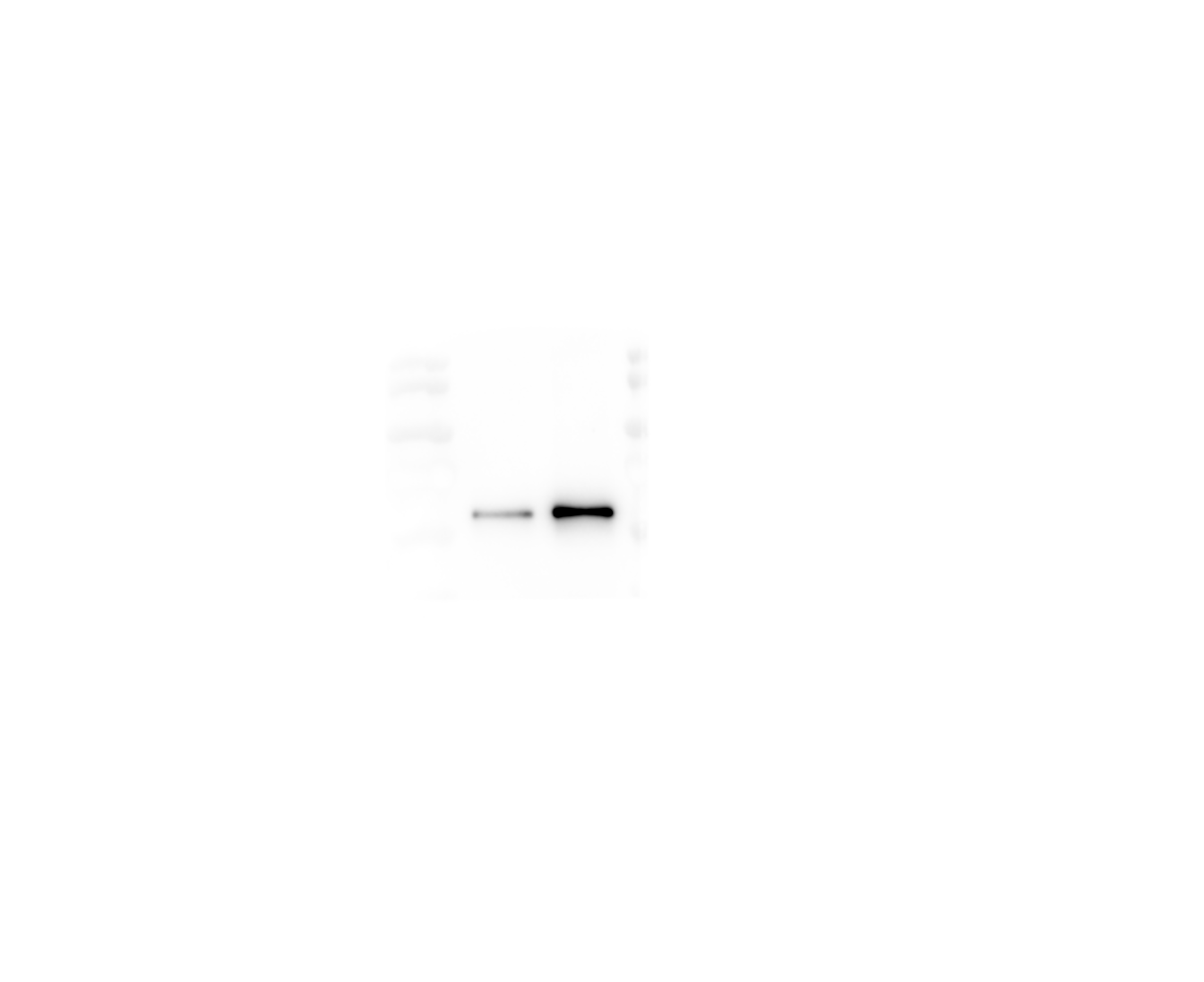

Supplement: Figure 1—figure supplement 1—source data 2. [file elife-98166-fig1-figsupp1-data2.zip › Supplementary Figure1G-pull-down (HA)-source data.Tif]

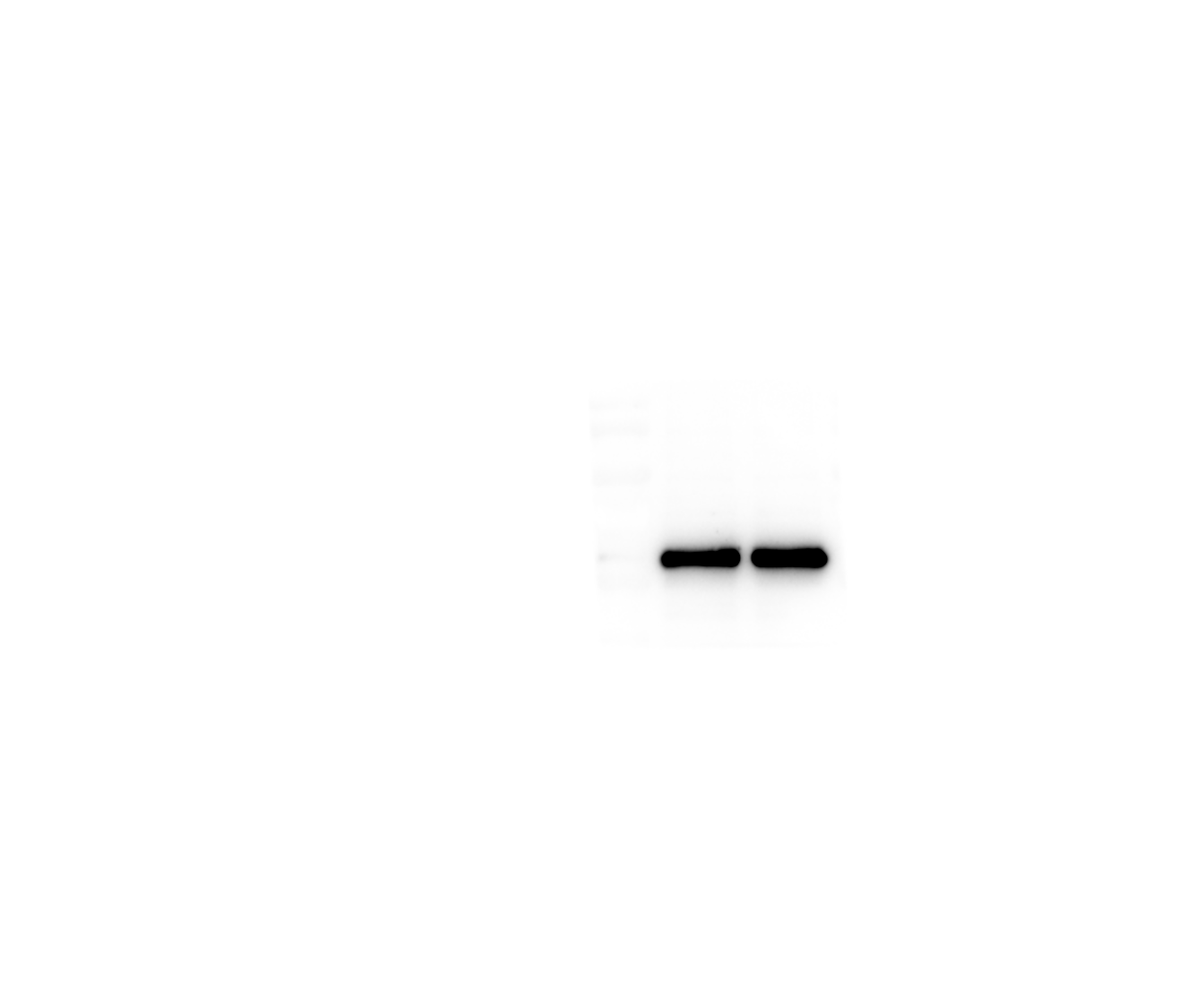

Supplement: Figure 1—figure supplement 1—source data 2. [file elife-98166-fig1-figsupp1-data2.zip › Supplementary Figure1H-input∩╝êHA∩╝ë-source data.Tif]

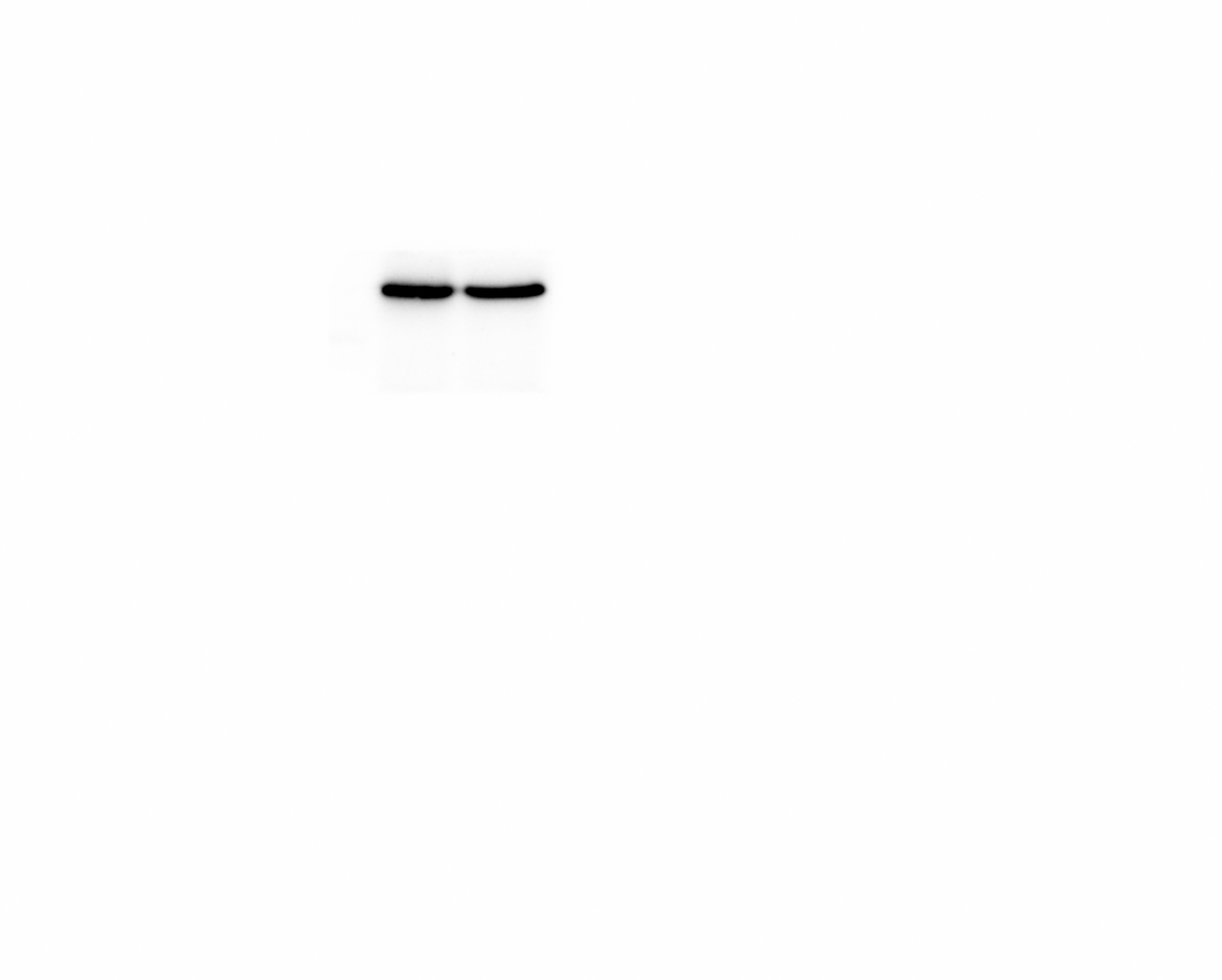

Supplement: Figure 2—source data 2. [file elife-98166-fig2-data2.zip › Western blot analysis of the GAPDH protein original file shown in Figure 2F..Tif]

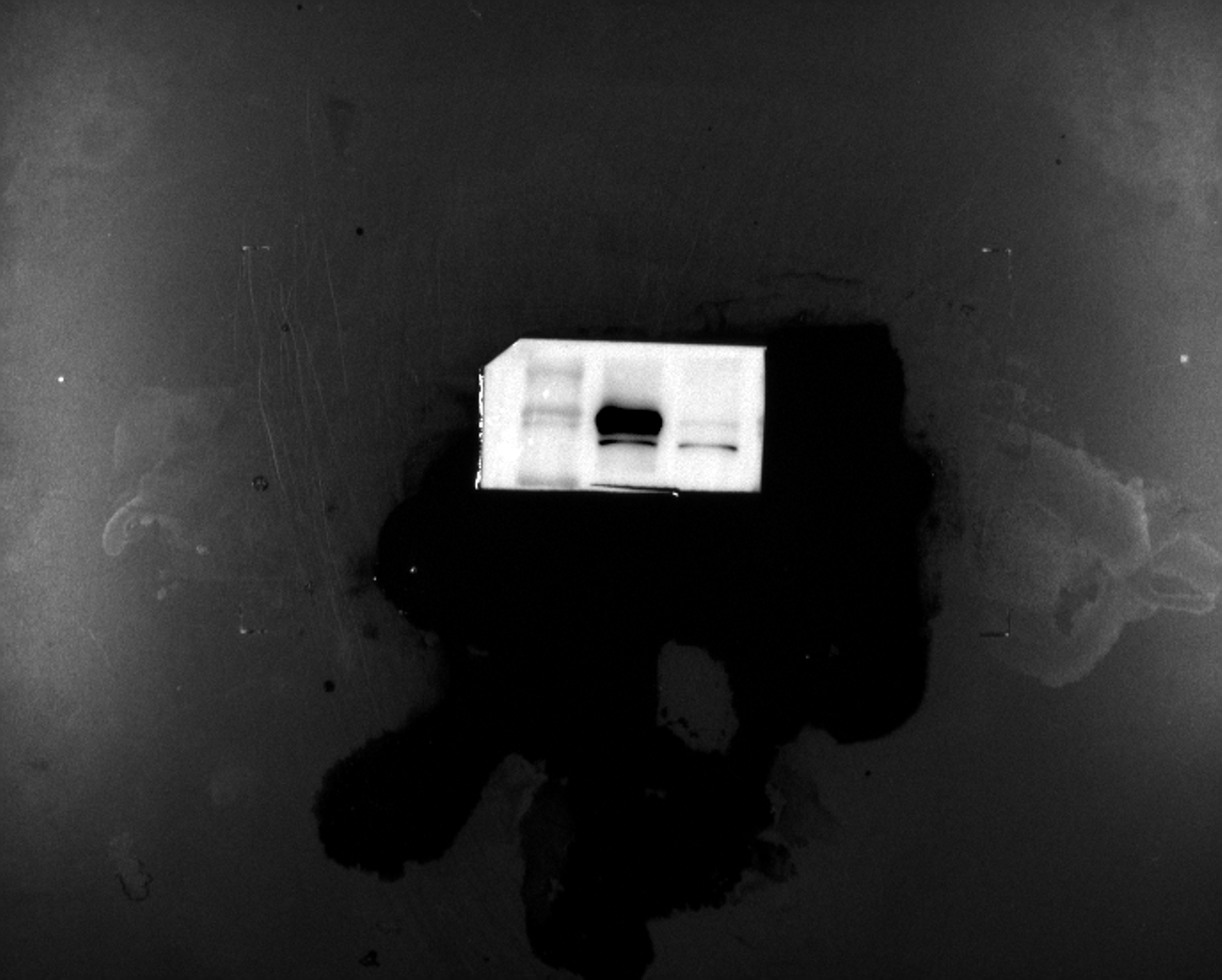

Supplement: Figure 2—source data 2. [file elife-98166-fig2-data2.zip › Western blot analysis of the NSUN2 protein original file shown in Figure 2E.Tif]

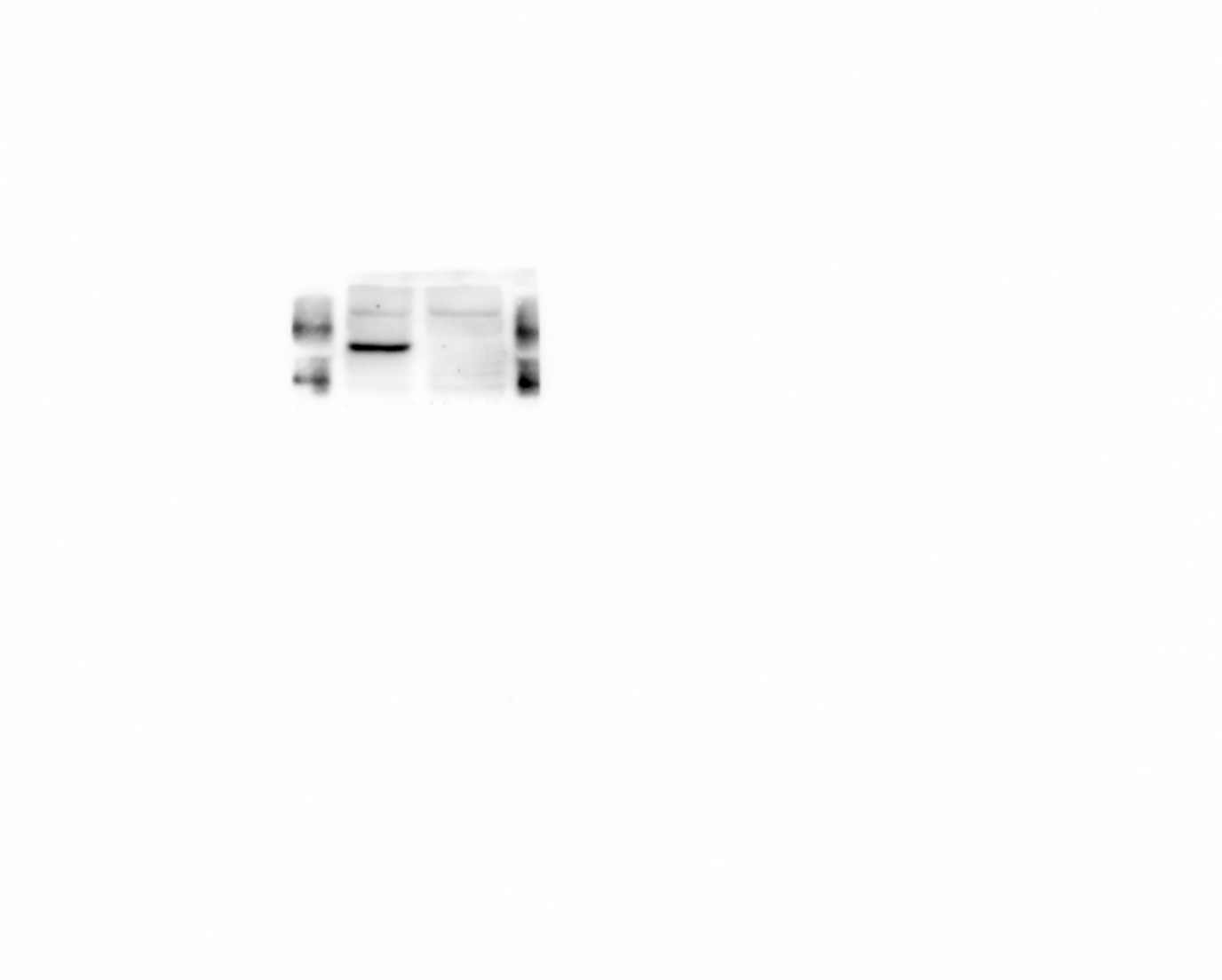

Supplement: Figure 2—source data 2. [file elife-98166-fig2-data2.zip › Western blot analysis of the NSUN6 protein original file shown in Figure 2F.Tif]

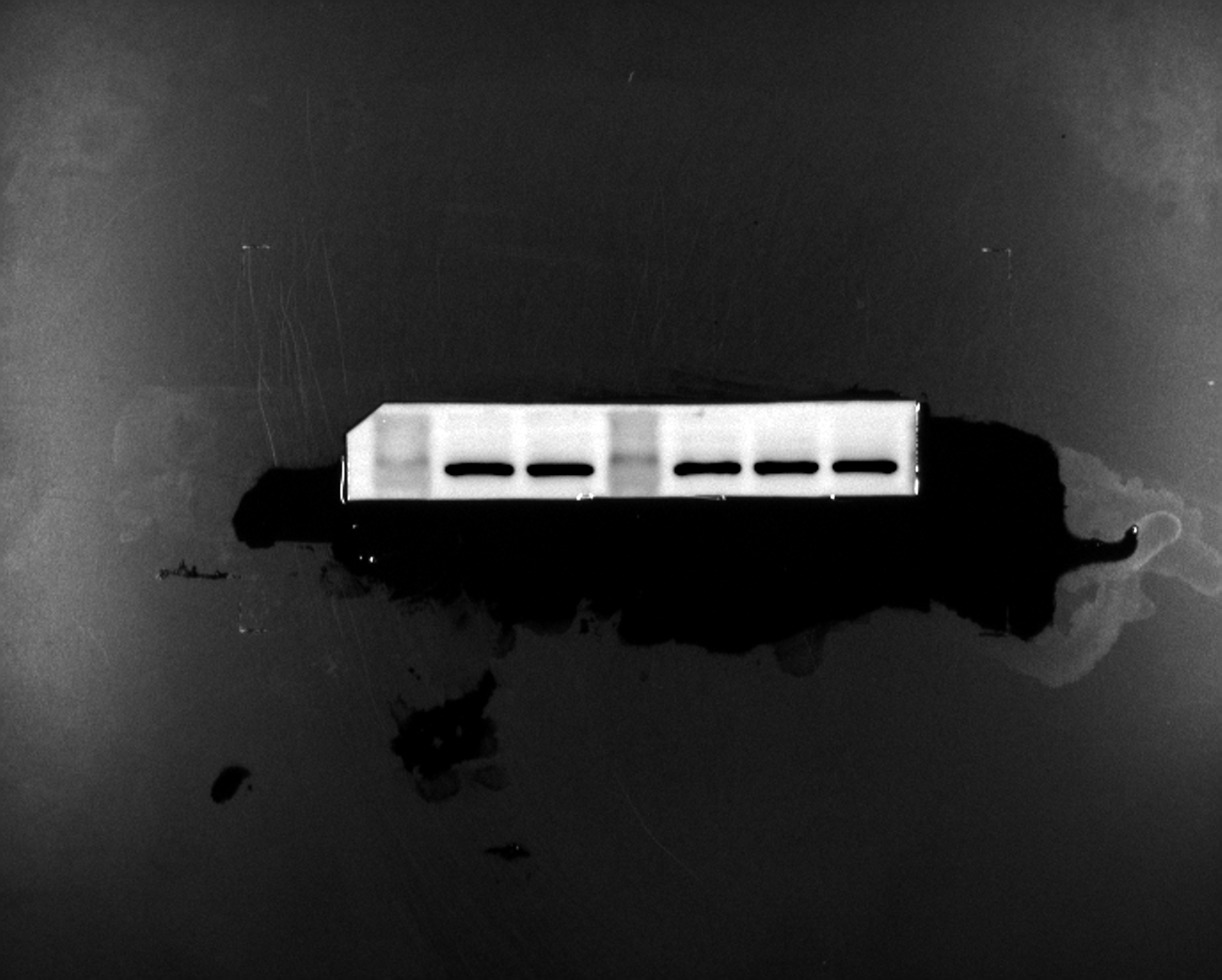

Supplement: Figure 2—source data 2. [file elife-98166-fig2-data2.zip › Western blot analysis of the ╬▒-Tubulin protein original file shown in Figure 2E..Tif]

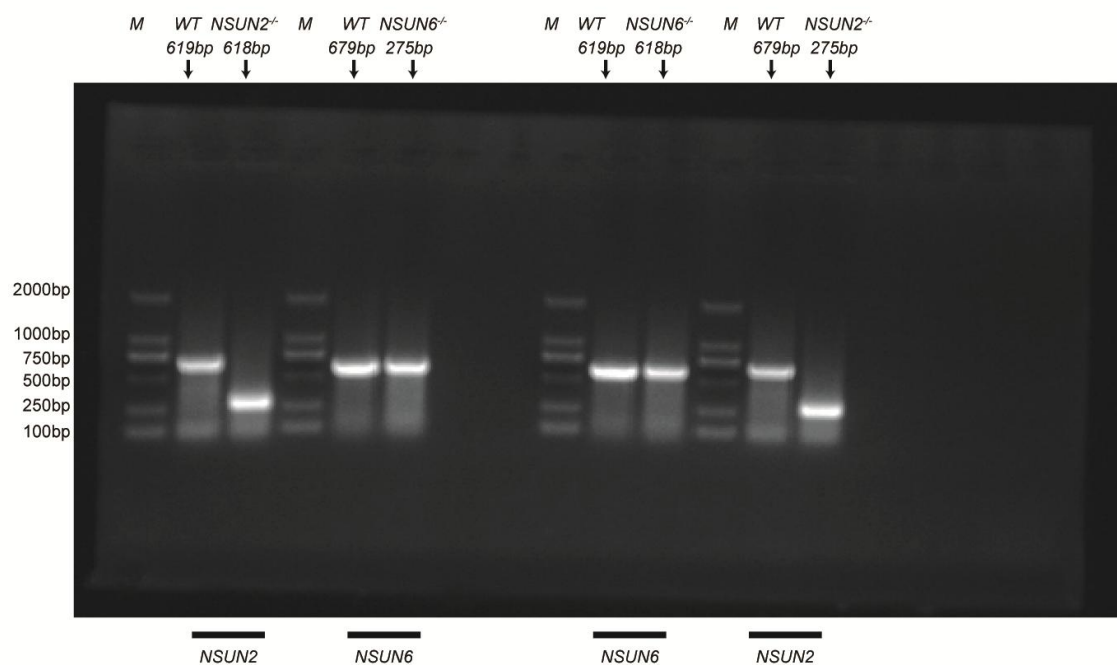

Supplement: Figure 2—figure supplement 3—source data 1. [file elife-98166-fig2-figsupp3-data1.zip › Figure 2-figure supplement 3B-source data1.pdf]

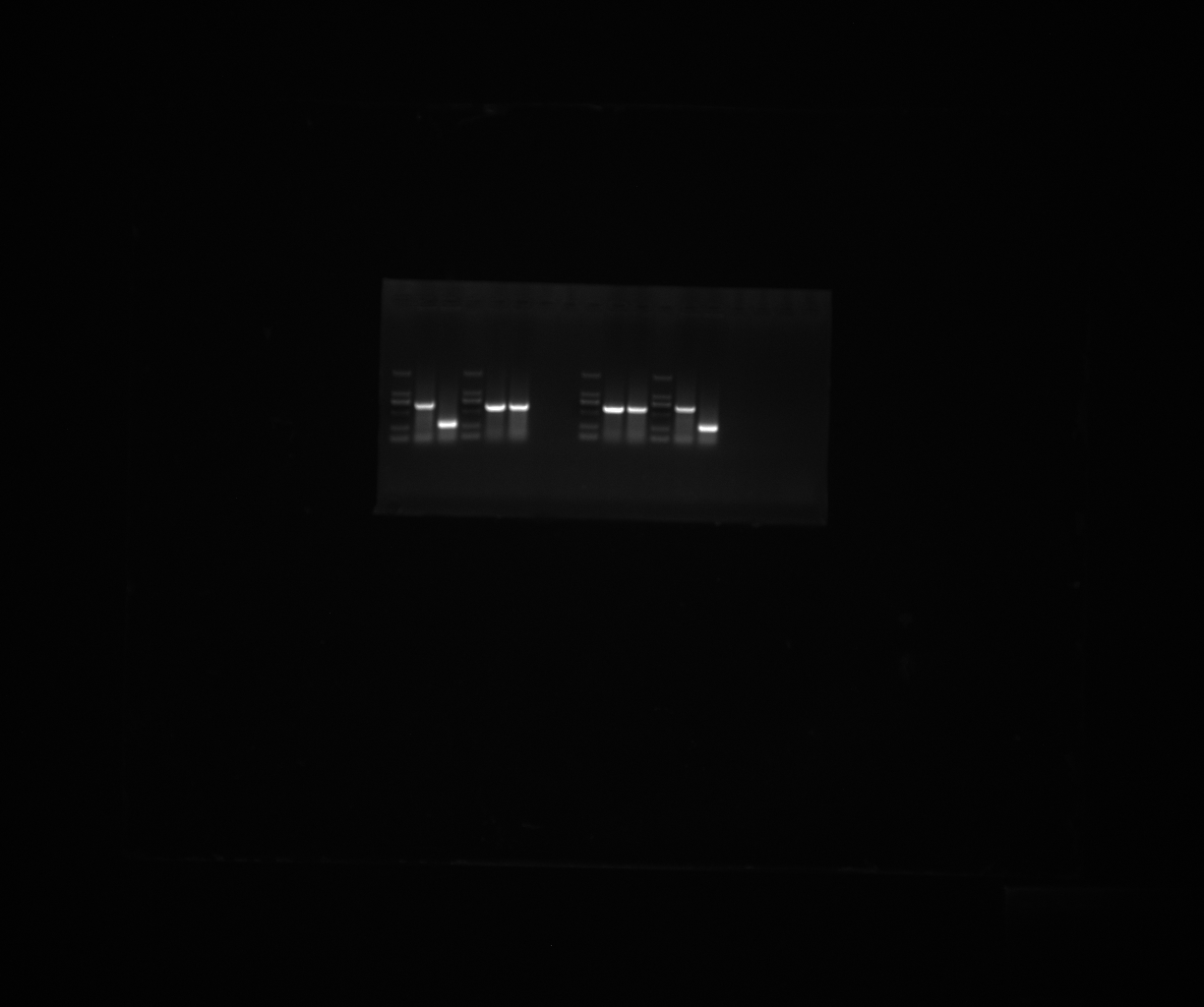

Supplement: Figure 2—figure supplement 3—source data 2. [file elife-98166-fig2-figsupp3-data2.zip › Figure 2-figure supplement 3B-source data2.tif]

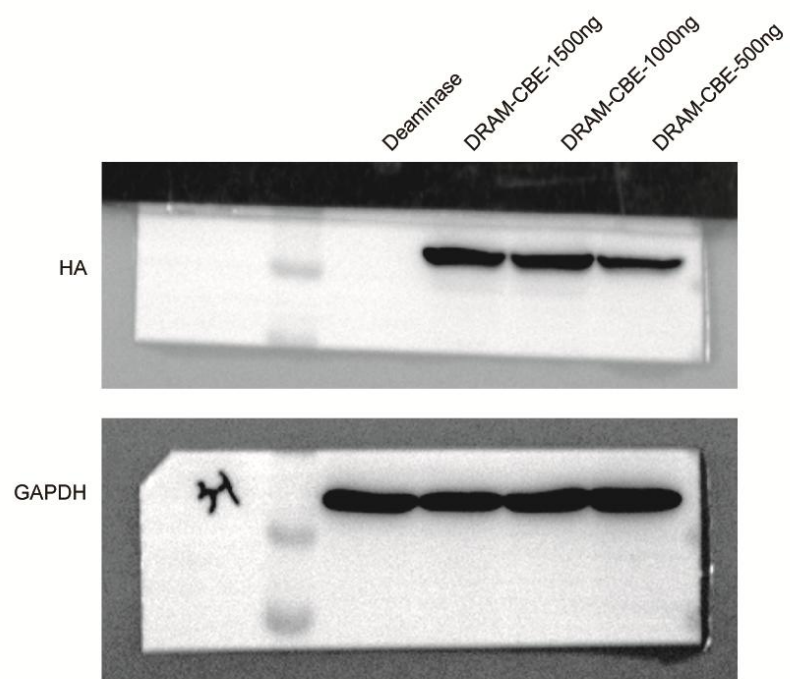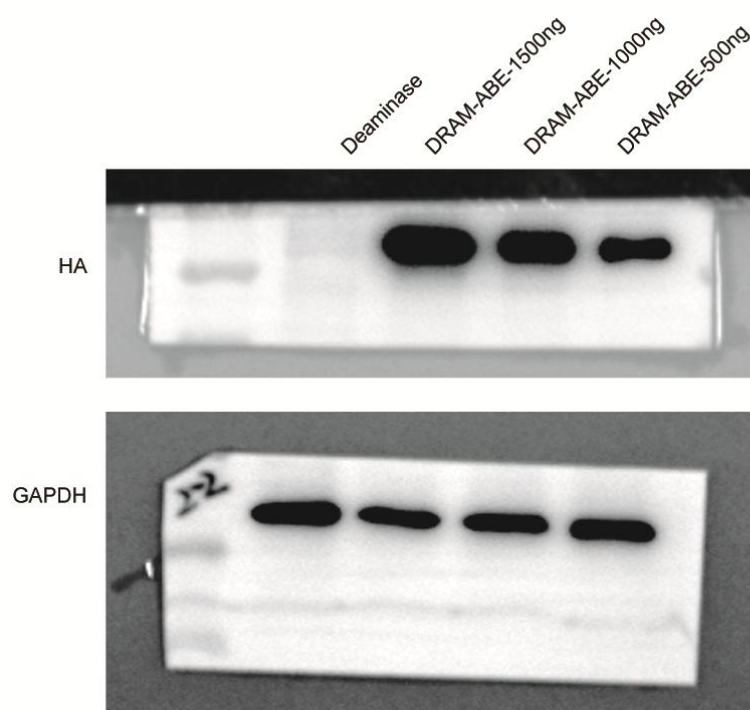

Supplement: Figure 5—source data 1. [file elife-98166-fig5-data1.zip › Figure 5-source data1/Figure 5-source data1.pdf]

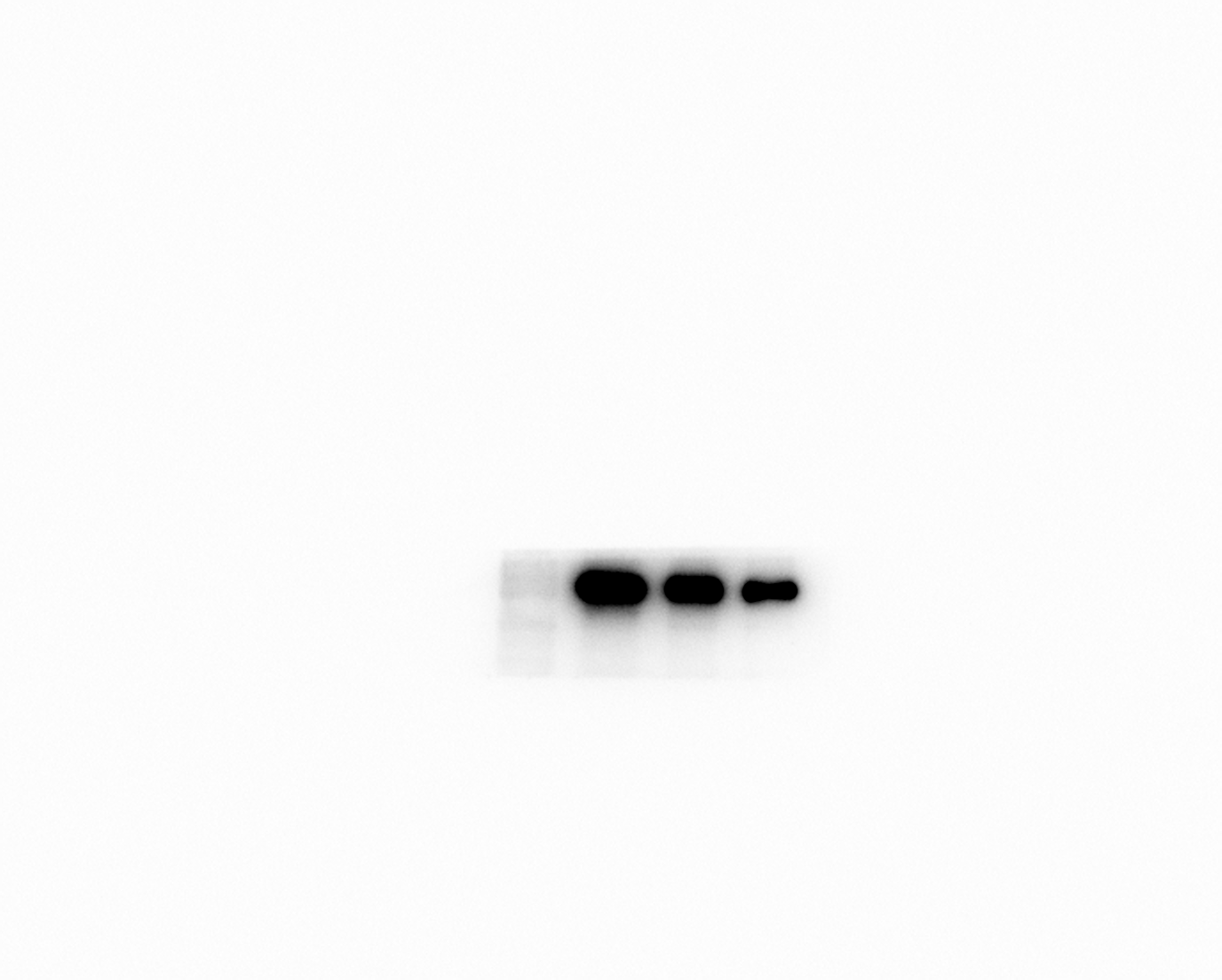

Supplement: Figure 5—source data 2. [file elife-98166-fig5-data2.zip › Western blot analysis of the HA protein original file shown in Figure 5F..tif]

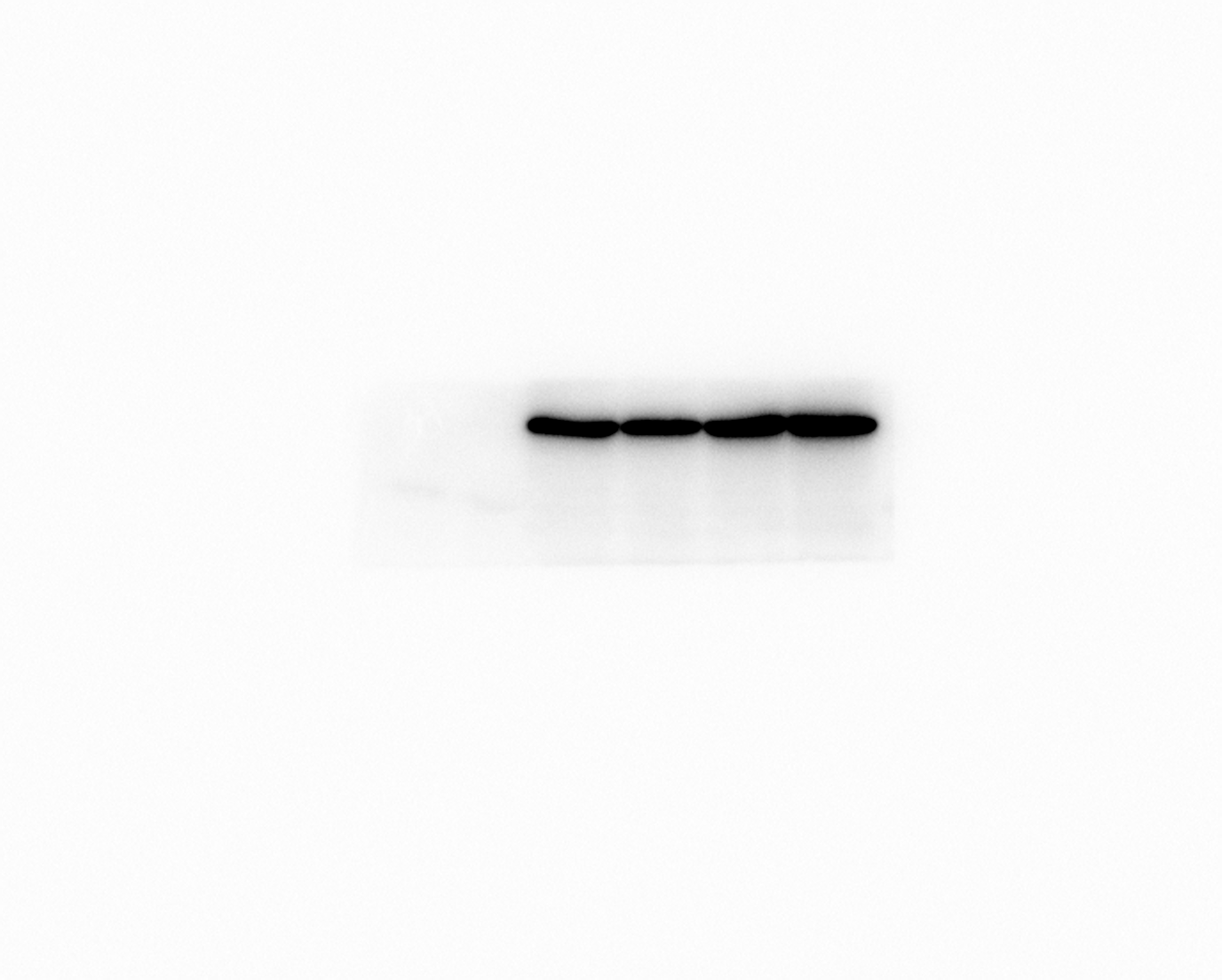

Supplement: Figure 5—source data 2. [file elife-98166-fig5-data2.zip › Western blot analysis of the GAPDH protein original file shown in Figure 5E..tif]

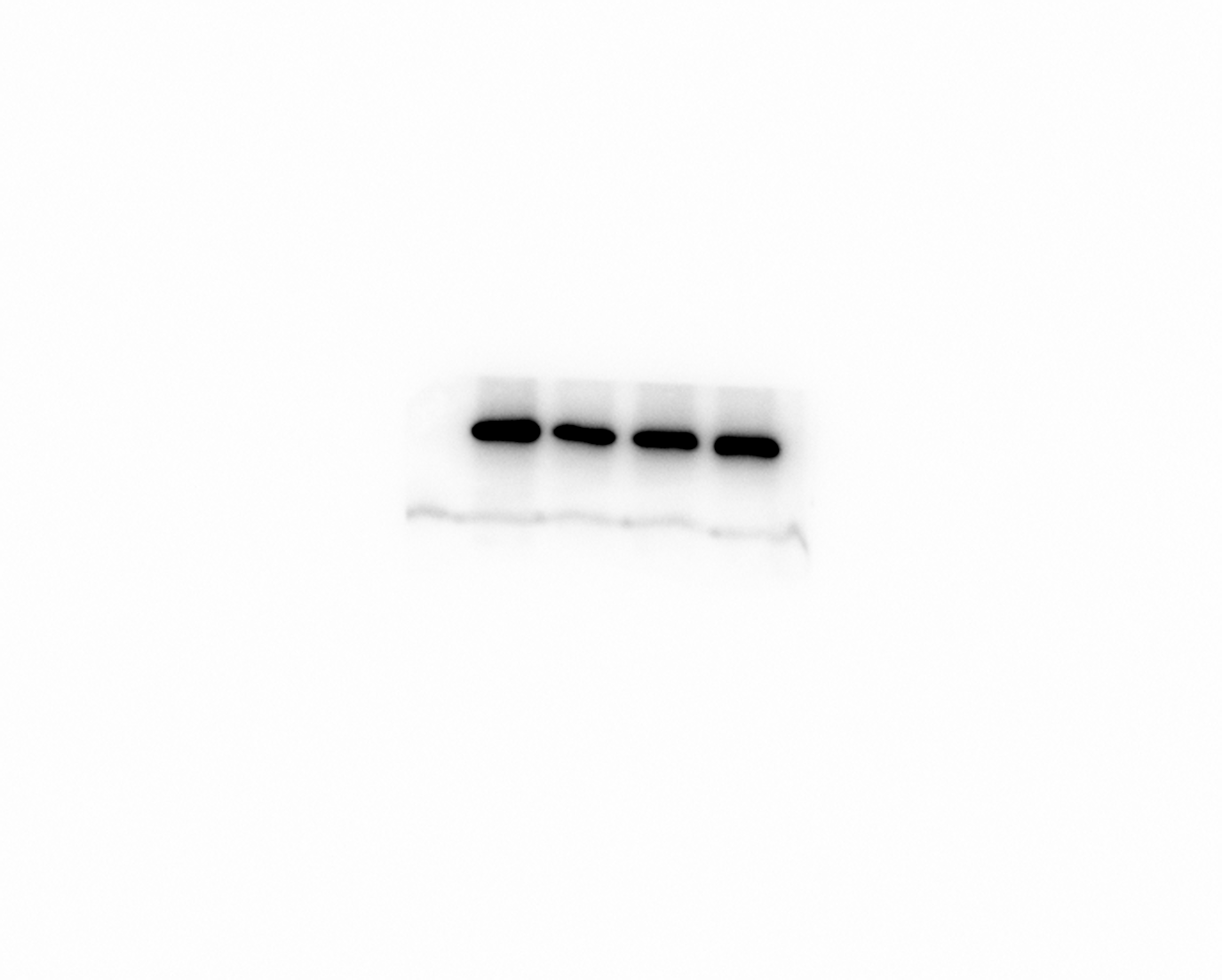

Supplement: Figure 5—source data 2. [file elife-98166-fig5-data2.zip › Western blot analysis of the GAPDH protein original file shown in Figure 5F..tif]

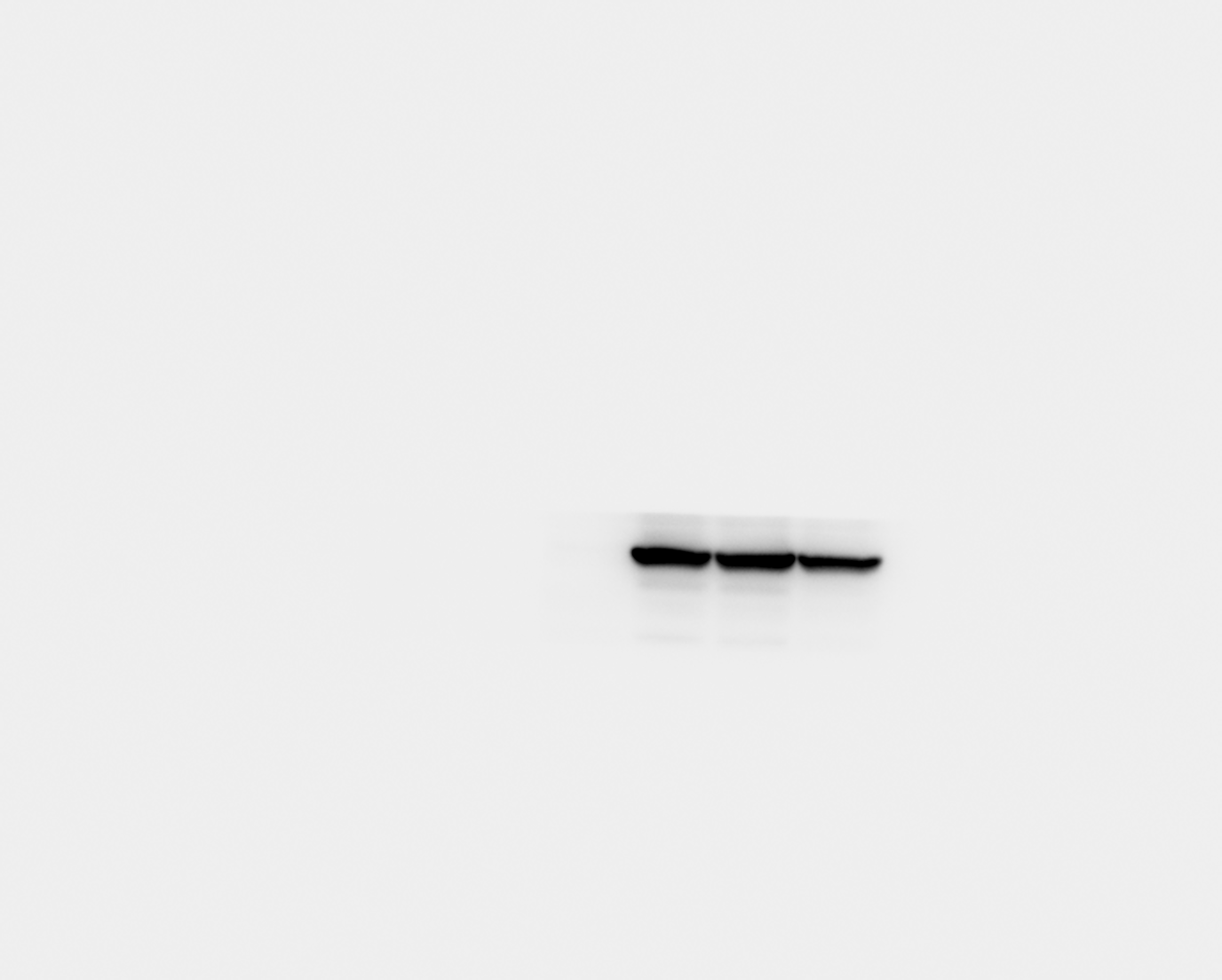

Supplement: Figure 5—source data 2. [file elife-98166-fig5-data2.zip › Western blot analysis of the HA protein original file shown in Figure 5E..tif]
