# Supplementary material for: Transcriptome-wide identification of 5-methylcytosine by deaminase and reader protein-assisted sequencing
Source: eLife. 2025 Apr 8;13:RP98166. doi: 10.7554/eLife.98166 (PMC11978299; doi:10.7554/eLife.98166)
Supplement: Figure 2—source data 1. [file elife-98166-fig2-data1.zip › Figure 2-source data1/Figure 2- Source Data 1.pdf]

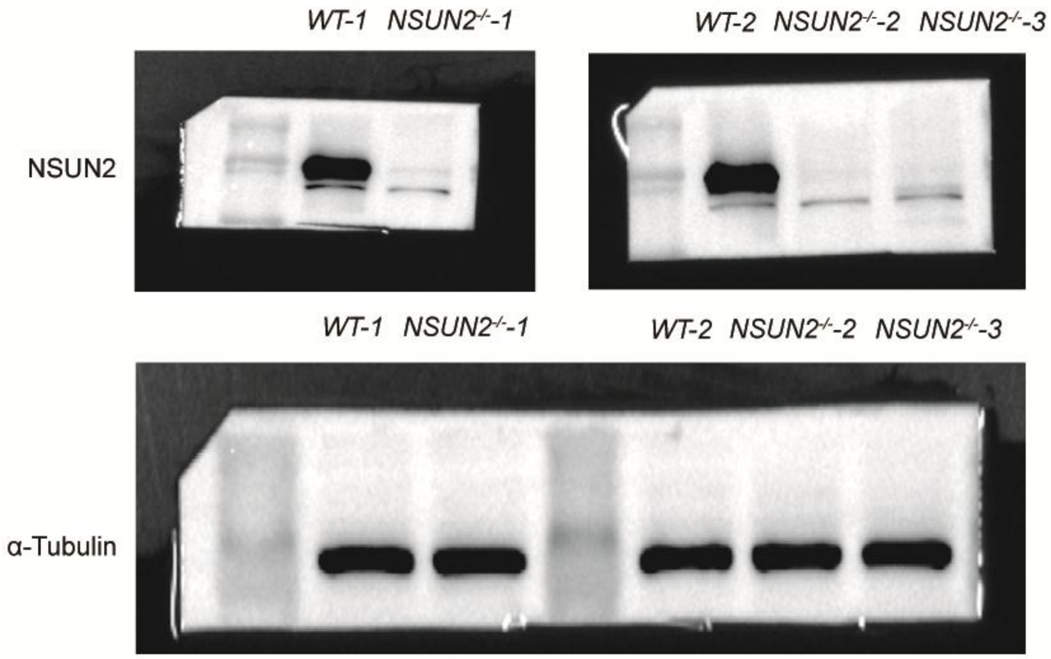

Note: the original western blots for Figure 2E are the WT-1 and NSUN2$^{-/-}$-1 samples in the above figure.

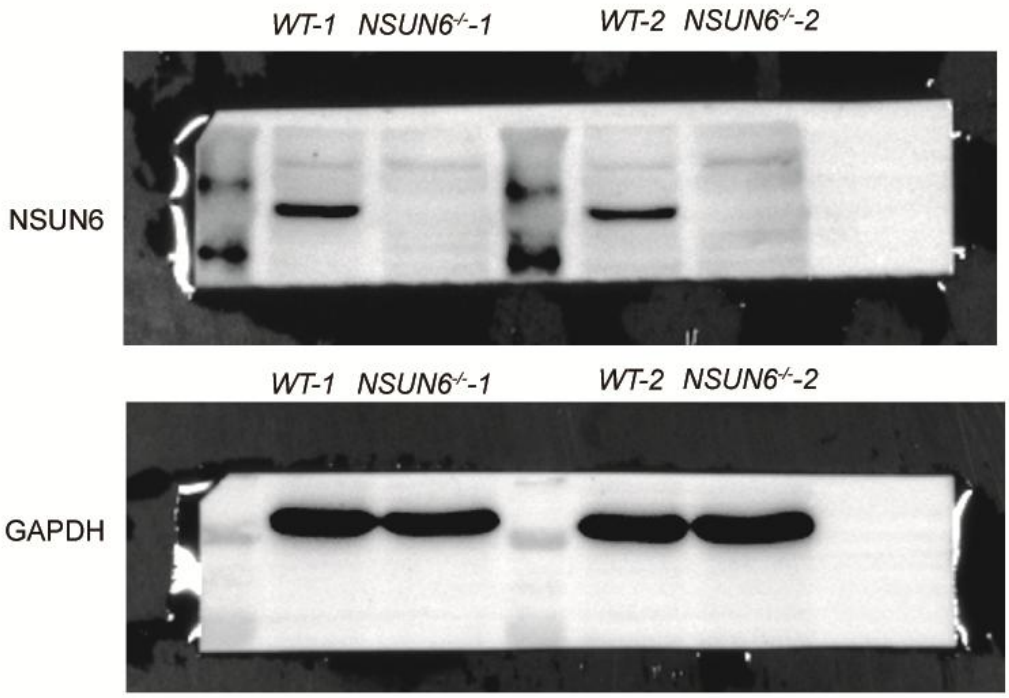

Note: the original western blots for Figure 2F are the WT-1 and NSUN6$^{-/-}$-1 samples in the above figure.
