# Supplementary material for: Transcriptome-wide identification of 5-methylcytosine by deaminase and reader protein-assisted sequencing
Source: eLife. 2025 Apr 8;13:RP98166. doi: 10.7554/eLife.98166 (PMC11978299; doi:10.7554/eLife.98166)
Supplement: Supplementary file 1. [file elife-98166-supp1.docx]

**Supplementary File 1: The detection for m^5^C in RNA.**

| **Detection method** | **Advantages** | **Disadvantages** |
| --- | --- | --- |
| BS-seq | Single nucleotide resolution;  Primitive methylation patterns. | Influenced by the secondary structure of RNA;  Disability for distinguishing m^5^C from other type of cytosine modifications,  Causes RNA damage. |
| m^5^C-RIP-seq | Distinguishable between m^5^C and other types of cytosine modifications. | Insensitive to low-abundance m^5^C;  Influenced by the secondany structure of RNA;  Relatively low esolution;  Highly dependent on antibody specificity. |
| miCLIP-seq | Specific detection of the m^5^C sites of NSUN2 methyltransferase. | Unable to detect m^5^C sites of other methyltransferases;  Highly dependent on antibody specifity;  Altered methylation patterns;  Time-consuming; Expensive. |
| AZA-lP-seq | MethyItransferase-specific detection of m^5^C;  Single nucleotide resolution. | Highly dependent on antibody specificity and 5-azaC binding efficiency;  High toxiciticy of 5-azaC. |
| TAWO-seq | Single nucleotide resolution;  Non-methylated cytosines have a low false positive rate of conversion. | Unstable conversion efficiency;  Dependent on the oxidation efficiency of peroxotungstate. |
| Nanopore-seq | Raw DNA or RNA can be directly sequenced. | Expensive; High error rate;  Sequencing signals probably affected by multiple nucleotides at the same time. |
