# Supplementary material for: Transcriptome-wide identification of 5-methylcytosine by deaminase and reader protein-assisted sequencing
Source: eLife. 2025 Apr 8;13:RP98166. doi: 10.7554/eLife.98166 (PMC11978299; doi:10.7554/eLife.98166)
Supplement: Supplementary file 4. — (a) The primers for vector construction. (b) The primers used for genotyping. (c) Single guide RNA. (d) The primers for DRAM-Sanger analysis. (e) The primers for qPCR. (f) The primers for bisulfite-sequencing PCR. [file elife-98166-supp4.docx]

**Supplementary File 4**

**Supplementary File 4a: The primers for vector construction**

| **Name of Primer** | **Sequences (5′−3′)** |
| --- | --- |
| ABE-ALYREF-ALYREF-Fwd  ABE-ALYREF-ALYREF-Rev  ABE-ALYREF-TadA-8e-Fwd  ABE-ALYREF-TadA-8e-Rev  ABE-YBX1-YBX1-Fwd  ABE-YBX1-YBX1-Rev  ABE-YBX1-TadA-8e-Fwd  ABE-YBX1-TadA-8e-Rev  CBE-ALYREF-ALYREF-Fwd  CBE-ALYREF-ALYREF-Rev  CBE-ALYREF-APOBEC1-Fwd  CBE-ALYREF-APOBEC1-Rev  CBE-YBX1-YBX1-Fwd  CBE-YBX1-YBX1-Rev  CBE-YBX1-APOBEC1-Fwd  CBE-YBX1-APOBEC1-Rev | agcagcggggggtcaatgcccgattccgcg  ttttgagccgccagaactggtgtccattctcgcat  tctggcggctcaaaaagaacc  tgaccccccgctgctg  agcagcggggggtcaatgagcagcgaggccgagacccagc  ttttgagccgccagactcagccccgccctgc  tctggcggctcaaaaagaacc  tgaccccccgctgctg  gagtccgccacaccaatgcccgattccgcg  gtaggggtactcgagactggtgtccattctcgcattatagg  ctcgagtacccctacgacgtg  tggtgtggcggactctg  gagtccgccacaccaatgagcagcgaggccgagac  gtaggggtactcgagctcagccccgccctg  ctcgagtacccctacgacgtg  tggtgtggcggactctg |
| YBX1^mut^-Fwd | ttgtacaccagactgccataaggaagtaccttcgcagtgt |
| YBX1^mut^ -Rev | acactgcgaaggtacttccttatggcagtctggtgtacaa |
| ALYREF ^mut^-Fwd | gccctgaaggccatggcgcagtacaacggcgt |
| ALYREF ^mut^ -Rev | acgccgttgtactgcgccatggccttcagggc |

**Supplementary File 4b: The primers used for genotyping**

| **Name of Primer** | **Sequences (5′−3′)** |
| --- | --- |
| NSUN2-KO -PCR-Fwd | ccccttagagctgttcgctgt |
| NSUN2-KO -PCR-Rev | gtcgaagaaaacaccgtcgcctt |
| NSUN6-KO -PCR-Fwd | taccatgttgaagcccaagaa |
| NSUN6-KO -PCR-Rev | gaagctactaaggcccagttt |

**Supplementary File 4c: Single guide RNA**

| **Name of Primer** | **Sequences (5′−3′)** |
| --- | --- |
| NSUN2-PX459sgRNA1-H-Fwd | caccgcgccatcctccgcgtcctc |
| NSUN2-PX459sgRNA1-H-Rev | aaacgaggacgcggaggatggcgc |
| NSUN2-PX459sgRNA2-H-Fwd | caccgggtggtggaaagcgcggcg |
| NSUN2-PX459sgRNA2-H-Rev  NSUN2-PX459sgRNA3-H-Fwd  NSUN2-PX459sgRNA3-H-Rev  NSUN2-PX459sgRNA4-H-Fwd  NSUN2-PX459sgRNA4-H-Rev  NSUN6-PX459sgRNA1-H-Fwd  NSUN6-PX459sgRNA1-H-Rev  NSUN6-PX459sgRNA2-H-Fwd  NSUN6-PX459sgRNA2-H-Rev | aaaccgccgcgctttccaccaccc  caccgaggctaccccgagatcgtca  aaactgacgatctcggggtagcctc  caccgtgttctccttgacgatctcg  aaaccgagatcgtcaaggagaacac  cacctaggtaaacaagaagcagaa  aaacttctgcttcttgtttaccta  caccatttttcacatgttgtactg  aaaccagtacaacatgtgaaaaat |

**Supplementary File 4d: The primers for DRAM-Sanger analysis**

| **Name of Primer** | **Sequences (5′−3′)** |
| --- | --- |
| RPSA-DRAM-Few | gcaaatgaaggaggaggatgt |
| RPSA-DRAM-Rev | gttagtgaaggttccaggagtg |
| AP5Z1-DRAM-Few | agggacttcggtgcagatta |
| AP5Z1-DRAM-Rev | ctcaagcctccaatcagagc |
| tRNA-Val-DART- Few | gtttccgtagtgtagtgg |
| tRNA-Val-DART-Rev | ctcaactggtgtcgtggagtcggcaattcagttgagtggtgtttccgccc |
| tRNA-Lys-DART- Few | gcccggctagctcagt |
| tRNA-Lys-DART-Rev | ctcaactggtgtcgtggagtcggcaattcagttgagtggcgcccaacgtg |
| tRNA-Asp-DART- Few | tcctcgttagtatagtg |
| tRNA-Asp-DART-Rev | ctcaactggtgtcgtggagtcggcaattcagttgagtggtggctccccgt |
| 28S rRNA4417-DART- Few | ggtacacctgtcaaacggtaa |
| 28S rRNA4417-DART-Rev | ccaagcacatacaccaaatgtc |
| 28S rRNA3782-DART- Few | cagccgacttagaactg |
| 28S rRNA3782-DART-Rev | cctcccacttattctacac |

**Supplementary File 4e: The primers for qPCR**

| **Name of Primer** | **Sequences (5′−3′)** |
| --- | --- |
| NSUN2-H-qPCR-Fwd | atcttgagaaaatcgccacac |
| NSUN2-H-qPCR-Rev | atcattcgcaataacaaatccct |
| NSUN6-H-qPCR-Fwd | tcagcgtgatcggcaagatt |
| NSUN6-H-qPCR-Rev  GAPDH-H-qPCR-Fwd  GAPDH-H-qPCR-Rev | acctaaagcagtcacaatctcct  gtctcctctgacttcaacagcg  accaccctgttgctgtagccaa |

**Supplementary File 4f: The primers for bisulfite sequencing PCR**

| **Name of Primer** | **Sequences (5′−3′)** |
| --- | --- |
| RPSA-BS-Fwd | tgttgttattgaaaattttgttgatg |
| RPSA-BS-Rev | ataatcaaccctaaaatcaataacca |
| AP5Z1-BS-Fwd | gttagtttgttattgaggttaggtt |
| AP5Z1-BS-Rev | ctcaactcaatattacctaaaaacaaa |
